# Therapeutic Interventions of Gut-Brain Axis as Novel Strategies for Treatment of Alcohol Use Disorder Associated Cognitive and Mood Dysfunction

**DOI:** 10.3389/fnins.2022.820106

**Published:** 2022-02-02

**Authors:** Xin Li, Le-Mei Chen, Gajendra Kumar, Shan-Jin Zhang, Quan-hai Zhong, Hong-Yan Zhang, Guan Gui, Lv-Le Wu, Hui-Zhen Fan, Jian-Wen Sheng

**Affiliations:** ^1^Department of Gastroenterology, The People’s Hospital of Zhangshu City, Jiangxi, China; ^2^Department of Pharmacy, Guangdong Provincial People’s Hospital Ganzhou Hospital, Ganzhou Municipal Hospital, Jiangxi, China; ^3^Department of Neuroscience, City University of Hong Kong, Kowloon Tong, Hong Kong SAR, China; ^4^Department of Gastroenterology, The People’s Hospital of Yichun City, Jiangxi, China; ^5^School of Chemistry and Bioengineering, Yichun University, Jiangxi, China

**Keywords:** alcohol use disorder (AUD)s, brain-gut axis, cognitive impairment, hippocampus-amygdala- frontal cortex circuit, mood disorder

## Abstract

Alcohol use disorders (AUD) is characterized by persistent or intermittent alcohol cravings and compulsive drinking. The functional changes in the central nervous system (CNS) after alcohol consumption are alcohol-associated cognitive impairment and mood disorders, which are major health issues reported in AUDs. Studies have shown that transferring the intestinal microbiota from AUDs patients to germ-free animals causes learning and memory dysfunction, depression and anxiety-like behavior, indicating the vital role of intestinal microbiota in development of neuropsychiatric disorders in AUD. Intestinal flora composition of AUD patients are significantly different from normal people, suggesting that intestinal flora imbalance orchestrate the development of neuropsychiatric disorders in AUD. Studies suggests that gut microbiome links bidirectional signaling network of the enteric nervous system (ENS) to central nervous system (CNS), forming gut-microbe-brain axis (brain-gut axis). In this review, we discussed pathogenesis and possible treatment of AUD-induced cognitive deficits, anxiety, and depression disorders. Further, we described the mechanism of intestinal flora imbalance and dysfunction of hippocampus-amygdala-frontal cortex (gut-limbic circuit system dysfunction). Therefore, we postulate therapeutic interventions of gut-brain axis as novel strategies for treatment of AUD-induced neuropsychiatric disorders.

Alcohol use disorder (AUD) is one of the most prevalent mental disorders worldwide. AUD causes a high disease burden as about 3.3 million deaths are attributing to AUD worldwide each year (WHO, 2014). The AUD is characterized by persistent or intermittent alcohol cravings and compulsive drinking. Symptoms of alcohol withdrawal are anxiety, sleep disturbance, headache, nausea, hallucinations, delirium, and epilepsy drinking (WHO, 2014). Long-term alcoholism and withdrawal in AUD patients cause cognitive impairment and emotional changes, which are manifested as deficits in acquisition, consolidation or retrieval of memory, depression, and anxiety (Stavro et al., 2013). Additionally, alcohol alters the composition and function of intestinal flora by affecting the metabolism, immunity, and intestinal barrier of the host, leading to the disturbance of colo-intestinal flora (Leclercq et al., 2014; Jansen et al., 2015; Fan et al., 2018; Bajaj, 2019). Interestingly, transplants of gut microbes from AUDS patients or alcohol-fed mice into normal healthy controls significantly changed the composition of gut bacteria, shifted behavioral phenotypes, and exhibited cognitive and mood impairments as reported in AUD patients (Bercik et al., 2011; Zhao et al., 2020). It suggests that alterations in the composition and abundance of intestinal flora directly correlate with patient’s cognitive function and emotional changes. The gut microbiome plays a key role in shaping social behavior patterns.

Intestinal flora are microbes living in the gut, includes not only bacteria, but also Eukarya, Archaea (Gill et al., 2006). Gut microbiota is considered as independent organ system (Cryan and Dinan, 2012; Clarke et al., 2014; Liu, 2016) to perform specific and vital functions. The intestinal flora participates in nutrition, digestion, and immunity. It also plays a vital role in brain’s behavioral and cognitive functions. Bidirectional communication between brain and gut are well known for past two decades. The intimate communication between the intestinal microbiome and host CNS is one of the key mechanisms for development of several mental disorders. Studies have shown that alcohol-induced intestinal flora imbalance could influence the patient’s cognitive function, mood change, and drinking behavior through the interactions with the immuno-endocrine system and vagus nerve. Therefore, dissecting the potential mechanism of gut-brain communication is crucial for treatment strategies of AUD-induced neuropsychiatric disorders (Figure 1). In this review, we described the pathogenesis, possible treatment strategies of AUD-induced cognitive deficits, anxiety, depression, and pathogenesis of intestinal flora imbalance. This review provides a new sight and strategies for treating AUD-induced neuropsychiatric disorders.

**FIGURE 1:**
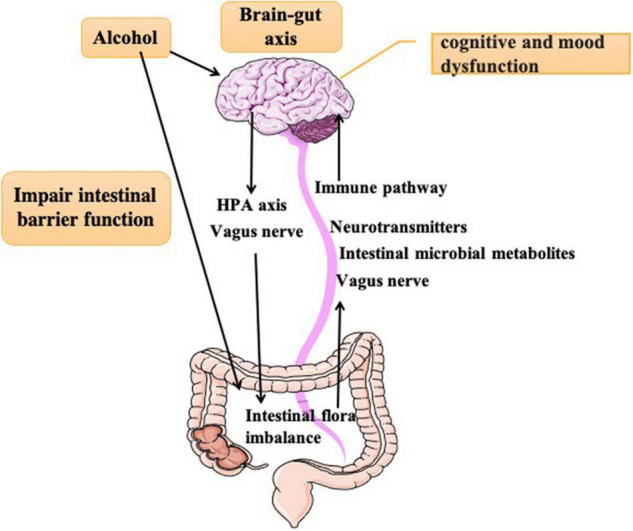
Alcohol directly acts on the brain to cause cognitive and emotional dysfunction and causes intestinal flora disorder through the vagus mechanism and HPA axis. In turn, intestinal flora disorder acts on the brain through the vagus nerve and immune endocrine pathway to cause behavioral abnormalities.

## Alcohol Use Disorders Related Neuropsychiatric Disorders

### Alcohol Use Disorders Induced Depression and Anxiety

Repetitive and high-frequency drinking causes alteration in CNS function and induce mental disorders, such as depression and anxiety. AUD is often accompanied by sleep disturbance, headache, hallucinations, delirium, depression, and anxiety. These symptoms in turns affects each other in diagnosis and treatment. The dysfunction of emotional control centers are associated with limbic system including the prefrontal cortex, amygdala, and hippocampus circuits (Witkiewitz et al., 2019). Studies have shown that acute alcohol intake activates brain neurotransmitter receptors, such as GABA and dopamine receptors, and produces anti-anxiety behavior temporarily; while chronic alcohol dependence inhibit GABA receptor activity, which offsets the anti-anxiety effect induced by acute alcohol intake and produces anxiety symptoms (Bruce et al., 2005; Sharma et al., 2007). Repeated withdrawal of alcohol drinking leads to over-activation of CNS, which makes alcohol-dependent patients more susceptible to mood disorder, resulting in negative emotions after stopping drinking (Kushner et al., 2000). Long-term alcohol intake and withdrawal in AUD are prone to emotional disorders such as depression and anxiety.

The abundance of Firmicutes decreased in AUD patients, while Bacteroidetes and Proteobacteria are increased significantly (Fan et al., 2018). The changes of intestinal microflora in patients with depression and anxiety has been reported as similar to the intestinal flora imbalance induced by alcohol (Jiang et al., 2015; Chen et al., 2019). We believe that alcohol-induced intestinal microflora imbalance is one of the causes of mood disorder. Studies reports that transplantation of the fecal microbiota from alcohol-exposed mice to the recipients can transfer the behavioral phenotype of chronic alcohol use disorder to the recipients, who manifested similar depression, anxiety and alcohol seeking behaviors (Bercik et al., 2011; Xiao et al., 2018; Zhao et al., 2020). Zhao et al. (2020) suggests that when germ-free Swiss Webster mice with fresh fecal contents are colonized by microbial flora of BALB/c mice, these original germ-free Swiss Webster mice showed similar anxiety-like behavior to those of BALB/c mice. In contrast, germ-free BALB/c mice colonized with the microbiota of Swiss Webster mice showed reduction in anxiety-like behavior and similar phenotype to the behavior of Swiss Webster mice separated into two groups, one of the groups was transplanted with fecal microbiota from the patients with alcoholism and marked as FMT-Alc, while other group transplanted with the fecal microbiota from the alcohol-free adults is marked as FMT-Con (Zhao et al., 2020). In the open field test, the average time spent in the central area of test chamber in FMT-Alc mice was significantly less than FMT-Con, indicating that mice transplanted with the intestinal flora from patients with alcoholism show more anxiety-like behavior than those transplanted with intestinal flora of non-drinking adults (Zhao et al., 2020). The findings were further supported by elevated plus maze (EPM) test, where percentage of time spent in open arms of FMT-Alc mice was significantly decreased as compared to FMT-Con mice. In tail suspension test (TST), the immobility time of FMT-Alc mice was significantly increased than FMT-Con mice, suggesting that the intestinal flora of mice receiving fecal microbiota transplantation from alcohol patients can promote depression-like behaviors (Zhao et al., 2020). Current animal experiments have described the correlation between alcohol and mood disorders through the brain-gut axis and the possible mechanisms and treatments. There are very few clinical studies, and there are differences between humans and animals. External factors such as disease factors in human body and living environment may lead to different responses to alcohol. Secondly, the limited number of experimental animals in the study may lead to errors in the results. Therefore, more clinical studies are needed to confirm the validity of this idea.

These studies support the hypothesis that the gut microbiota affects emotional behaviors, possibly *via* the gut-microbe-brain axis (or brain-gut axis). The regulation from the bottom-up signaling, through the vagus nerve and immune pathways, will affect behaviors, brain activities, and levels of neurotransmitters and their receptors and neurotrophic factors. The CNS signals will affect intestinal functions, intestinal permeability, and change in composition of intestinal microbiota through a top-down approach. The list of studies related to mood disorder as shown in Table 1.

**TABLE 1:** Pre-clinical studies related to mood disorder due alteration of gut flora.

	Study type	Major points			References
Changes in intestinal flora	Pre-clinical	The abundance of Bacteroidetes and Proteobacteria in AUD patients increased significantly, which are similar to those seen in patients with mood disorders.			Fan et al., 2018
Mimicking the behavioral phenotype of chronic alcohol	Pre-clinical	Transplantation of the fecal microbiota from alcohol-exposed mice manifested depression, anxiety behaviors	FMT-Alc altered gut microbiota structure of recipients	Erysipelotrichia, Erysipelotrichaceae, Erysipelotrichales, Bacteroides, Parabacteroides, and Alloprevotella was increased Lactobacillaceae, Lactobacillus, Lactobacillales, and Bacilli was decreased	Xiao et al., 2018
			(a) Anxiety-like and depression-like behaviors (b) Decreased social interaction behaviors (c) Spontaneous alcohol preference (d) Decreased BDNF level in brain		Zhao et al., 2020

### Alcohol Use Disorders Induced Memory and Cognitive Impairment

Alcohol use disorder is linked to widespread cognitive deficits (Stavro et al., 2013). Long-term excessive drinking causes a wide range of neurological diseases, including cognitive and learning and memory deficits (Liappas et al., 2007; Jansen et al., 2015; Manning et al., 2016; Gass et al., 2017). AUD patients often show deficits in identifying novel objects and initiates sporadic memory impairment at early stage (Kraynak et al., 2018). In the free recall test, AUD patients lose the ability to remember words clearly (Cerqueira et al., 2007). However, no significant impairment was observed in the memory of the same object in the implicit assessment of backward reading or word completion tasks (Cerqueira et al., 2007) suggesting that influence of alcohol on memory tasks is selective. Additionally, alcohol intake causes long-term deficits in recognizing the novel object (Chandler et al., 2017). In middle stages of AUD, patients may lose their spatial memory (Bowden and McCarter, 1993). Spatial learning and memory are mainly regulated by cortex and hippocampus (Hermann et al., 2014; Marin et al., 2017; Schwarzmeier et al., 2019). Wei et al. (2019) reported that alcohol increases the permeability of blood-brain barrier (BBB). It causes neuronal loss in hippocampus and cortex, leading to disordered arrangement and distribution, and even degeneration and necrosis of the neurons (Wei et al., 2019). The metabolic activity in the neurons of hippocampus decreases, resulting in impairment of spatial cognition after alcohol abuse. Animal studies suggest that the acquisition of spatial learning, memory and the execution of working memory tasks are impaired in the novel object location test due to damage of hippocampus neurons (Santin et al., 2000). Primate study (ale rhesus monkeys) reports that chronic administration of alcohol for prolonged time require more training as compared to control groups to reach the average cognitive performance in visuospatial memory tasks. These animal studies showed that AUD increases the difficulty of learning the novel task and subsequent retention of spatial memory task (Crean et al., 2011). These outcomes are consistent with the results from previous clinical studies performed on teenage female AUD patients with intermittent alcohol abuse. Teenagers showed impaired spatial learning and consolidation of memory (Caldwell et al., 2005; Contreras et al., 2019). The findings of pre-clinical and clinical studies suggest that long-term alcohol abuse impair the acquisition and execution of spatial memory tasks (Crean et al., 2011).

Alcohol affects cognitive function through direct and indirect pathways. Chronic drinking induces intestinal permeability and microbial imbalance through the brain-gut axis pathways, causing intestinal microbial metabolites to enter the blood and act on the frontal limb circuits (amygdala, hippocampus, hypothalamus, insula, etc.) Ventral striatum, medial prefrontal lobe, and pre-cingulate cortex) lead to an inflammation cascade, which affects memory by changing the structure and/or function of specific brain regions, such as the hippocampus and/or the medial prefrontal cortex Process. At the same time, immune cells in the intestine secrete significant amounts of cytokines in response to the imbalance of intestinal flora, which is transported to the brain through the bloodstream, aggravating memory deficits (Sherwin et al., 2019). Alcohol destroys the intestinal barrier and increase intestinal permeability due to direct cell damage (transepithelial mechanism). Alcohol intake causes change in the intestine, including mucosal ulcers, erosions, loss of epithelium mainly at the tip of the villi, and weakening of the cell membrane. In addition, reactive oxygen species (ROS) are released during alcohol metabolism, and the process of oxidative stress directly leads to cell damage. Another pathway involves the triggering of paracellular mechanisms. Alcohol and its metabolites act on the tight junction complexes of cells (redistribute proteins, destroy the tight junctions of adjacent cells; change the expression of tight junction proteins) to increase in the permeability of cells. Intestinal microbial metabolites enter the bloodstream and act on the frontal limbic circuits (amygdala, hippocampus, hypothalamus, insula, ventral striatum, medial prefrontal lobe, and pre-cingulate cortex) (Blakemore, 2008), leading to inflammatory responses in the brain-gut system and stress response (Hillemacher et al., 2018). The list of studies related to cognitive dysfunction as shown in Table 2.

**TABLE 2:** Cognitive dysfunction associated with AUD and alteration of gut flora.

	Stage pre-clinical/clinical	Major points	References
Novelty memory	Pre-clinical	Lack of microbes impairs social skills and is flawed in identifying social novelties	Kraynak et al., 2018
		Cognitive deficits in novelty caused by alcohol intake are long-term	Chandler et al., 2017
	Clinical	Intestinal flora imbalance can impair the subject’s ability in the free recall test	Witkiewitz et al., 2019
Spatial memory	Pre-clinical	Animals whose drinking causes hippocampus damage have impaired access to new locations	Santin et al., 2000
		Insufficient spatial memory in the alcohol dependent group	Bowden and McCarter, 1993
	Clinical	People with a history of alcoholism have deficits in spatial memory	Nagahara et al., 1995
		Teenagers with AUD exhibit obvious brain abnormalities during the completion of spatial memory tasks	Caldwell et al., 2005
		High-dose intermittent and excessive ethanol consumption impaired spatial learning and memory	Contreras et al., 2019

## The Signal Pathways Involved in Alcohol Use Disorders Related Neuropsychiatric Disorders

Chronic alcohol-induced neuropsychiatric disorders due to brain dysfunction has drawn research attention suggesting that intestinal microbiota plays a significant role in the anatomy, physiology, and immune host functions. It affects the brain and ultimately the nervous system through immune, endocrine, and vagus mechanisms.

### Immune Pathway and Alcohol Use Disorders

**FIGURE 2:**
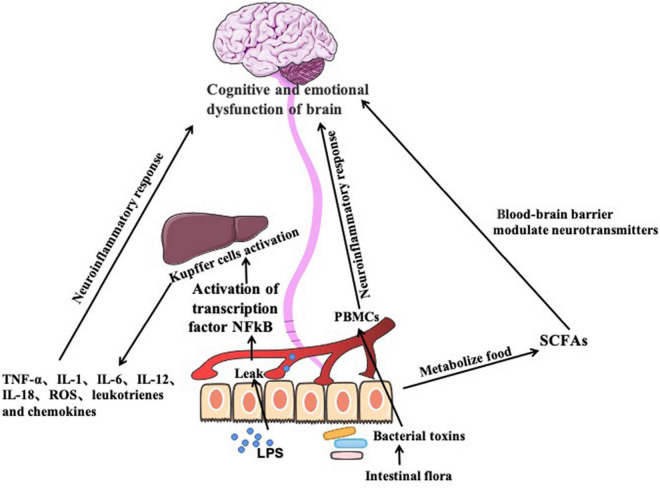
Imbalance of the gut flora induces production of proinflammatory factors through immune pathway, which causes neuroinflammatory response and cause cognitive and emotional dysfunction. Gut microbes also produce short-chain fatty acids, which are signaling molecules acting on central nervous system to cause cognitive and emotional dysfunction in the brain.

Excessive drinking changes the intestinal barrier function, resulting increased intestinal permeability, and bacterial translocation. The intestinal permeability is assessed by examining gram-negative enterobacteria lipopolysaccharide (LPS) antibodies in plasma (Akira and Hemmi, 2003; Gimenez-Gomez et al., 2019). Increased intestinal permeability due to overgrowth of gram-negative bacteria in the upper small intestine, results in leak of bacteria LPS to the circulation. It is an effective pro-inflammatory agent activating the transcription factor NFkB through the CD14-TLR4 receptor (Akira and Hemmi, 2003). NFkB activation induces Kupffer cells to produce pro-inflammatory chemokines, such as tumor necrosis factor-a (TNF-α), interleukin-1(IL-1), interleukin-6(IL-6), and interleukin-12(IL-12), interleukin-18(IL-18), reactive oxygen species (ROS), leukotrienes and chemokines. Along with lipopolysaccharides, the pro-inflammatory cytokines are sequestered to brain regions for induction of neuroinflammatory responses. Activation of peripheral blood mononuclear cell (PBMCs) by gut-derived bacterial toxins contributes to inflammatory response. Expression and activation of LPS receptors such as TLR4 and CD14, and PGN receptor TLR2 in PBMCs of alcoholics are higher than healthy people (O’Brien et al., 2004; Evrensel et al., 2020). Another study suggests that depression is associated with a low-expression of inflammatory markers in the intestine, characterized by elevated levels of pro-inflammatory cytokines, such as IL1, IL-6, and TNF-a (Crews et al., 2006). These inflammatory mediators act on the brain-gut axis to produce depression and anxiety-like behaviors (Figure 2).

Recent studies also suggest that symbiosis of the intestinal flora produces endotoxins to induce inflammatory responses in the brain leading to cognitive impairment. This hypothesis is confirmed by increased level of Escherichia coli and LPS in the stool and blood samples of AD and mild cognitive impaired (MCI) patient (Zhan et al., 2016).

### Endocrine Pathway and Alcohol Use Disorders

#### Hypothalamic-Pituitary-Adrenal Axis

**FIGURE 3:**
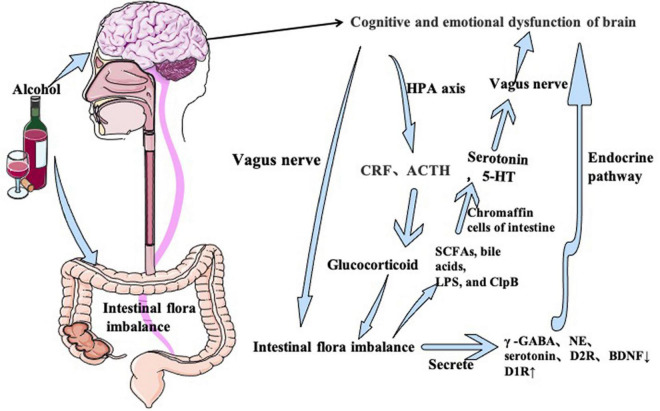
Alcohol can directly damage and affect the brain and induce cognitive and emotional dysfunction. At the same time, alcohol-induced brain injury can lead to intestinal microflora disorder through the vagus nerve and HPA axis. In addition, alcohol can directly act on the gut and cause intestinal flora disorder, and then produce neurotransmitters through intestinal flora’s own secretion and vagal nerve pathways, which in turn act on the brain to aggravate cognitive and emotional dysfunction.

HPA axis is the core regulatory system of stress responses in patients with chronic AUD (Scott et al., 2013; Moloney et al., 2014; Wang and Kasper, 2014; O’Mahony et al., 2017). In the brain-gut interactions, the HPA axis functions through endocrine pathway which belongs to the limbic system. Activation of the HPA axis increases secretion of corticotropin-releasing factor (CRF) in hypothalamus and adreno-cortico-tropic-hormone (ACTH) by the pituitary gland. ACTH triggers the release of the immunosuppressive stress hormones (cortisol in primates and corticosterone in rodents) at the adrenal cortex (Mawdsley and Rampton, 2005; Cussotto et al., 2018). Under the chronic stress of alcohol abuse for a long time, glucocorticoid release can increase intestinal mucosal barrier dysfunction (Yeager et al., 2011), leading to intestinal inflammation and changes in enteric microbiota. It causes cognitive changes due to alteration of intestinal flora. Stress-induced cortisol lead to abnormal intestinal permeability. Intestinal inflammation and changes in microbiota affect mood and cognitive function through the bidirectional regulation of the brain-gut axis (Figure 3).

#### Neurotransmitters

Intestinal bacteria secrete several neurotransmitters such as γ-GABA, norepinephrine (NE), serotonin (5-hydroxytryptamine), dopamine (Camilleri, 2009; Roshchina, 2016). It acts on host nerves to induce brain-derived neurotrophic factor (BDNF) expression, which activate or inhibit the CNS function. Abnormal intestinal flora in AUD patients lead disrupts the secretion of neurotransmitters and neurofactors, causing cognitive impairment, depression, and anxiety.

Decreased BDNF and dopamine receptor 2 (D2R) secretion (Berridge and Waterhouse, 2003; Hou et al., 2020) and increased dopamine receptor 1 (D1R) secretion (Jadhav et al., 2018) has been reported in patients with cognitive dysfunction. Levels of γ -GABA (Cryan and Kaupmann, 2005; Kumar et al., 2013; Olivier et al., 2013), serotonin (Wikoff et al., 2009; Hillemacher et al., 2018), norepinephrine (NE) (Diaz Heijtz et al., 2011; Clarke et al., 2013), and BDNF (aan het Rot et al., 2009; Diaz Heijtz et al., 2011; Gareau et al., 2011; Bus et al., 2015) are decreased in patients with depression and anxiety. These neurotransmitters are detected in CNS and produced by intestinal flora. GABA is produced by glutamate in food and broken down by lactobacillus and bifidobacterium in probiotics (Barrett et al., 2012). GABA- producing bacteria have been shown to relieve depression and anxiety behavior (Bravo et al., 2011; Strandwitz et al., 2019). Surprisingly, these phenomena were also observed in patients with AUD. Increased intestinal permeability induced by chronic alcohol abuse causes intestinal microflora disorder, leading to increased metabolism of tryptophan (a precursor of serotonin) to Kynurenine, which reduces serotonin synthesis and induces depression behavior (Hillemacher et al., 2018). Zhao et al. (2020) has reported decreased levels of BDNF, alpha 1 subunit of GABA type A receptor (α-1GABAAR) in the medial prefrontal cortex and decreased metabotropic glutamate receptors 1 in anxious mice. Therefore, alcohol induced-intestinal flora imbalance causes neurotransmitter metabolism disorder (Figure 3).

Notably, recent studies have found that tryptophan (TRP) metabolites through the Kynurenine pathway may play a significant role in psychiatric disorders (Wichers and Maes, 2004; Gimenez-Gomez et al., 2019; Leclercq et al., 2021). The metabolites mainly studied are Kynurenine (KYN), Kynurenic acid (KYNA), and quinoline acid (QUIN), among which KYNA and QUIN have neuroprotective effects and neurotoxicity, respectively. TRP was negatively correlated with depression score, elevated KYN levels were associated with the persistence of depression, elevated QUIN levels can produce locally inflammatory cytokines that have a detrimental effect on cognition (Gimenez-Gomez et al., 2019; Leclercq et al., 2021). Most of the peripheral tryptophan enters the Kynurenine pathway under the action of two rate-restriction enzymes, liver tryptophan 2, 3-dioxygenase (TDO) and extrahepatic indoleamine 2, 3-dioxygenase (IDO), and is gradually metabolized into Kynurenine, KYNA and QUIN (Gimenez-Gomez et al., 2019; Leclercq et al., 2021). Rate-limiting enzyme activity is affected by inflammatory stimuli such as cytokines, Toll-like receptor ligands, bacterial metabolites and bacteria-derived reactive oxygen species (O’Connor et al., 2009; Dantzer et al., 2011; Orhan et al., 2016). Chronic alcohol intake leads to increased intestinal permeability, LPS leakage induces IDO activation, and prolonged alcohol withdrawal leads to TDO activation in the liver, thereby enhancing the Kynurenine pathway, resulting in increased KYN and QUIN, decreased KYNA (Gimenez-Gomez et al., 2019; Leclercq et al., 2021), and ultimately decreased 5-hydroxytryptamine synthesis, which leads to behavioral and cognitive changes. In addition, the tryptophan metabolic pathway is directly or indirectly affected by intestinal bacteria and their metabolites, and the levels of circulating TRP, KYN, and 5-HT in GF mice were normalized after transplantation of normal intestinal flora to normal mice (1, Gimenez-Gomez et al., 2019).

#### Intestinal Microbial Metabolites

Intestinal microbial metabolites are signaling molecules acting on gut microbes and affects CNS (Erny et al., 2015). It includes short chain fatty acids such as propionate, acetate and butyrate. Several intestinal bacteria have high metabolic capacity and produce short-chain fatty acids (SCFA) by metabolizing food, which act on the brain through the blood-brain barrier and subsequently affects cognitive function and mood. Majority of the metabolites have anti-inflammatory properties to interact with the host’s immune system. SCFAs modulates the release of important neurotransmitters like enteroendocrine serotonin (5-HT) secretion (Holzer et al., 2012). Intestinal short-chain fatty acids are metabolized by intestinal bacteria such as clostridium, Bacteroides, propionibacterium, bifidobacterium, lactobacillus, eubacterium, Prevotella, and Rosicella (Macfarlane and Macfarlane, 2012).

Alcohol disrupts the balance of intestinal flora (Dalile et al., 2019). SCFAs acts directly on the CNS and influence disease and behavior by regulating epigenetic, neuroplasticity and gene expression (Dalile et al., 2019). Schroeder et al. (2007) reported that BDNF are affected by exogenous short-chain fatty acids (sodium butyrate) in the short term. A significant reduction in depression-like behavior was observed in mice receiving sodium butyrate over 28 days (Schroeder et al., 2007). These findings suggest that gut bacteria alter brain function by modulating neurotransmitters in the gut brain axis *via* microbial metabolites (Figure 2).

### Hippocampus-Amygdala-Cortex Frontal Limb Circuit and Alcohol Use Disorders

Alteration of microbial community and inflammatory response by alcohol use disorder affects the hippocampus-amygdala-cortex frontal limb circuit (Stavro et al., 2013; Jansen et al., 2015; Gass et al., 2017). Several studies have reported that depletion of microbiota in gut flora of mice exhibit deficits in emotional processing and cognitive function and abnormalities in different brain regions (Hsiao et al., 2013; Desbonnet et al., 2014; Luczynski et al., 2016a; Sgritta et al., 2019; Sherwin et al., 2019). The neuronal circuit including amygdala, hippocampus, hypothalamus, insula, ventral striatum, medial prefrontal lobe, and pre-cingulate cortex) orchestrates the peripheral inflammation and cognitive function (Schulte et al., 2012; Oscar-Berman et al., 2014; Alba-Ferrara et al., 2016). There are substantial evidence suggesting the long-term effect of alcohol consumption on learning and memory due to changes in the anatomical structure and/or function of brain regions (hippocampus and/or medial prefrontal cortex) (Beck et al., 2012). Amygdala development, HPA axis, hippocampal monoamine concentration, prefrontal cortex myelination, gene expression and dopaminergic neurotransmission in mesocortical circuits are regulated by the gut microbiota and its metabolites (Strandwitz, 2018; Stasi et al., 2019). The amygdala is a baroreceptor. It is located deep in temporal lobe of the brain and regulates negative emotions (Correia and Goosens, 2016; Cai et al., 2018; Sherwin et al., 2019). There are extensive functional association of amygdala with several brain regions through internal connections formed between subnuclei in the amygdala known as amygdala neural circuit. These complex neuronal circuit of amygdala regulates emotional responses. It has been reported that excessive activation of the amygdala causes mood disorders and cognitive dysfunction (Sripada et al., 2011; Aloi et al., 2018). The loss of the microbiota cause disturbance in amygdala neuronal circuit and leads to overall neurological hyperactivity (Luczynski et al., 2016b). Besides, the hippocampus is another important brain region related to brain-gut axis. The microbiota has been well-known to affect hippocampal’s neurogenesis and gene expression, thereby participating in neuroplasticity (Bercik et al., 2010; Stilling et al., 2014; Gronier et al., 2018). The hippocampus is major target of lipid metabolism (Faria et al., 2014; Oliveira et al., 2016). The brain-gut axis mediates the metabolism of glycerophospholipids in the hippocampus (Petra et al., 2015). Intestinal microbes regulate the neurobiochemistry of the brain *via* this pathway. It has also been reported that the neurobiological basis of mood disorders and cognitive functional changes includes the disorders of the neuroimmune and neuroendocrine systems, synaptic plasticity, impaired neurogenesis, and decreased hippocampal volume (Guida et al., 2018). The hippocampus is highly sensitive to environmental factors such as microbial composition (Mohle et al., 2016; Hueston et al., 2017). Studies have shown that microbial diversity, especially during specific fragile neurodevelopmental periods, may promote the restoration of hippocampal function (Bailey et al., 2011; Clarke et al., 2013). The inflammatory response caused by the gut microbiota causes significant decrease in hippocampal BDNF and monoamine neuromodulation, such as an increase in tryptophan and a decrease in Kynurenine, which lead to mood change and cognitive impairment (Desbonnet et al., 2015). Prefrontal cortex (PFC) regulates emotional functions and plays a vital role in the development of neuropsychiatric disorders (including depression, schizophrenia, autism, etc.) (Shallice and Cipolotti, 2018). It has been reported that myelination dynamics in prefrontal cortex is key factor for the pathogenesis of mental disorders (Bercury and Macklin, 2015. Therefore, we hypothesize that microbiota can be a potential therapeutic target for the treatment of mental disorder.

### The Vagus Nerve and Alcohol Use Disorders

The vagus nerve directly mediates intestinal permeability, immune-inflammatory response, endocrine signal transmission and intestinal reflex activities (Bonaz et al., 2018). Intestinal flora alters vagal nerve signals and causes behavioral abnormalities (Peirce and Alviña, 2019). It has been reported that specific bacterial strains utilize vagus nerve signals to communicate with brain and changes the behavior (Yu et al., 2020). Bravo et al. (2011) reposts that Lactobacillus rhamnosus treatment significantly decreased GABA mRNA levels in mice brain and reduce anxiety and depression-like behavior. However, these effects were not observed in vagus nerve-removed mice (Bravo et al., 2011). These finding suggests that imbalance of intestinal flora alteration caused by alcohol leads to mood disorders through the vagus nerve. Intestinal endocrine cells are main sensors of intestinal nutrients to interface intestinal contents with afferent nerves (Gribble and Reimann, 2016). Intestinal endocrine cells release a large number of gastrointestinal neurohormones, including cholecystokinin (CCK), glucagon-like peptide-1 (GLP-1), peptide YY (PYY), and serotonin, to regulate digestion, nutrient absorption and, food intake. Intestinal endocrine directly affect sensory neurons of vagus nerve *via* neurohormones (Bohorquez et al., 2015; Bellono et al., 2017; Kaelberer et al., 2018) diffusion to the neurons terminals (Williams et al., 2016). It has been reported that isolated nucleus tractus solitarius (NTS) is vital brain region to receive vagal nerve afferent signal, where visceral information is transmitted to cerebral cortex, rostral ventrolateral medulla, locus coeruleus (LC), hypothalamus, and dorsal raphe nucleus through the ascending neural pathway (Grill and Hayes, 2012; Hachem et al., 2018).

Dysregulation of intestinal flora increases the intestinal permeability. Intestinal microbiota produces a large number of metabolites, including short-chain fatty acids (SCFAs), cholic acids, lipopolysaccharides (LPS), and casein hydrolytic protease B (Heiss and Olofsson, 2018), which stimulates intestinal chromaffin cells and regulate serotonin biosynthesis (Yano et al., 2015). Metabolites of serotonin activates vagal afference *via* serotonin receptors (Williams et al., 2016). Recently, a neuronal circuit has been proposed to control learning and memory through the gut: the entero-vagus-NTS-medial-hippocampal pathway (Suarez et al., 2018). Suarez et al. (2018) reported that injury of vagus nerve or subphrenic nerve reduces hippocampal BDNF and impair the episodic memory in rats. Vagal dysfunction also induces depression-like behavior by affecting norepinephrine neurons in the locus coeruleus (LC) (Suarez et al., 2018; Figure 3).

The mechanisms underlying the potential link between the alcohol-induced intestinal flora imbalance and neuropsychiatric disorders have not been investigated widely and few AUD clinical trials study has been reported. There are more studies required to conclude that intestinal flora imbalance induced brain-gut axis dysfunction in alcohol use disorder are associated with cognitive impairment and mood disorder.

## Progress in the Treatment of Alcohol Use Disorders

AUD induced alteration of intestinal microflora by regulates brain function through immune, endocrine, vagus nerve, HAP axis, and other pathways (Ames et al., 2020; Nikolova et al., 2021). Therefore, the selection of therapeutic drugs for correcting intestinal microflora disorder may be a new therapeutic approach. However, the use of probiotics has been shown to restore the balance of gut flora (Ma et al., 2021). The commonly used probiotics are mainly Bifidobacterium, Lactobacillus, Clostridium.

### Bifidobacterium

In terms of negative emotions, Savignac et al. (2014) reported that Bifidobacterium Longum 1714TMM and Bifidobacterium Breve 1205 could reduce the depressive and anxious behaviors of mice. Bifidobacterium Breve 1205 induced low anxiety in elevated masts, while Bifidobacterium Longum 1714 induced antidepressant behavior in tail suspension tests (Savignac et al., 2014). Bifidobacterium longum NCC3001 reduced the depression scores of patients on the anxiety and depression Scale by more than 60% and weakened the response of several brain regions to negative emotional stimuli (Pinto-Sanchez et al., 2017). In terms of cognitive impairment, Bifidobacterium Breve A1 improved cognitive decline in elderly patients with mild cognitive impairment (MCI) and memory impairment in elderly patients (Kobayashi et al., 2019a,b). These studies indicate that bifidobacterium has a good effect on improving mood and cognitive function (Table 3).

**TABLE 3:** Therapeutic potential of bifidobacterium.

Flora species	Study type	Dosage and duration of treatment (Colony-forming units, CFU)	Result	Mechanism	References
Bifidobacterium longum 1714 tmm	Pre-clinical	1 × 10^9^ CFU 6 weeks	Reduces depression-like behavior	Immune pathway	Savignac et al., 2014
Bifidobacterium breve 1205	Pre-clinical	1 × 10^9^ CFU 6 weeks	Reduces anxiety-like behavior	Immune pathway	Savignac et al., 2014
Bifidobacterium longum NCC3001	Clinical	1 × 10^10^ CFU 6 weeks	Reduces depression-like and anxiety-like behavior	Unclear	Pinto-Sanchez et al., 2017
Bifidobacterium breve A1	Clinical	2.0 × 10^10^ CFU 24 weeks	Improved cognitive function	Unclear	Kobayashi et al., 2019a
Bifidobacterium breve A1	Clinical	2 × 10^10^ CFU 12 weeks	Improve memory disorders	Unclear	Kobayashi et al., 2019b

### Lactobacillus

In terms of negative emotions, Lactobacillus Casei Shirota and Lactobacillus rhmanosus JB-1 limits the depressive and anxiety-like behaviors (Bravo et al., 2011; Adikari et al., 2020). It was observed that Lactobacillus rhamnosus JB-1 improved depression and anxiety-related behaviors by regulating GABA synthesis (Bravo et al., 2011). Lactobacillus helveticus NS8 has an anti-anxiety effect by reducing serotonin levels (Luo et al., 2014). Lactobacillus plantarum 90sk and Bifidobacterium executing centis 150 reduced depressively-like behavior in forced swimming tests with an effect similar to fluoxetine (Yunes et al., 2020). Lactobacillus plantarum DR7 improves cognitive and memory dysfunction, and relieved anxiety symptoms (Chong et al., 2019). L. Plantarum C29 improved cognitive function, but there were no significant changes in TNF-α, IL-6, IL-1B, and cortisol concentrations in either the probiotic or placebo groups (Hwang et al., 2019). Lactobacillus Plantarum 299V improved cognitive performance and decreased KYN concentration through affecting Kynurenine pathway (Rudzki et al., 2019) (Table 4).

**TABLE 4:** Therapeutic potential of Lactobacillus.

Flora species	Study type	Dosage and duration of treatment (Colony-forming units, CFU)	Result	Mechanism	References
Lactobacillus plantarum 90sk and Bifidobacterium adolescentis 150	Pre-clinical	Respective 1 × 10^8^ CFU,1 × 10^7^ CFU, 2 weeks	Reduces depression-like behavior	GABA↑	Yunes et al., 2020
Lactobacillus casei Shirota	Clinical	3 × 10^9^ CFU over 8 weeks	Reduces depression-like and anxiety-like behavior	Unclear	Adikari et al., 2020
Lactobacillus helveticus NS8	Pre-clinical	1 × 10^9^ CFU/ml, According to daily amount of water consumed. 4 weeks	Reduces anxiety-like behavior	Serotonin ↑	Luo et al., 2014
Lactobacillus rhamnosus JB-1	Pre-clinical	1 × 10^9^ CFU 4 weeks	Reduces depression-like and anxiety-like behavior	Synthesis and regulation of GABA	Bravo et al., 2011
Lactobacillus plantarum DR7	Clinical	0.5 × 10^9^ CFU 12 weeks	Improved cognitive function, Reduces anxiety-like behavior	Serotonin ↑	Chong et al., 2019
Lactobacillus Plantarum 299v	Clinical	20 × 10^9^CFU 8 weeks	improved cognitive performance and decreased KYN concentration	Kynurenine pathway	Rudzki et al., 2019
Lactobacillus plantarum c29	Clinical	1 × 10^9^CFU/ml, 12 weeks	Improved cognitive function	BDNF↑	Hwang et al., 2019

### Clostridium

Clostridium is less studied than Bifidobacterium and Lactobacillus, however, the therapeutic potential has been reported. Several studies have reported that Clostridium butyricum MIYAIRI 588 is effective in treating refractory major depression when used in combination with antidepressants (Miyaoka et al., 2018). This is consistent with the results of Akkasheh et al. (2016) and Goh et al. (2019). However, other studies provide a different opinion, suggesting that probiotic therapy is only effective for patients with mood disorders such as mild or moderate depression (Ng et al., 2018).

These studies have reported that probiotic therapy shows great potential, but the results have not reached consensus, which may be caused by different strains, administration time, administration dose, and administration population. In addition, the probiotic mixture also showed a different effect, and we assume that they complement each other and have a better effect on regulating brain function. Pre-clinical studies have reported the efficacy, however, multi-center clinical studies are needed to investigate its effects.

## Conclusion

Alcohol abuse causes intestinal microbial imbalance, impairing CNS function resulting in cognitive dysfunction and mood changes. Evidence of probiotics treatment efficacy from animal models studies have established a close association of AUD-induced neuropsychiatric disorders with brain-gut axis, however, there are only few clinical studies to support this phenomenon. Clinical studies have been reported that alcohol causes abnormalities in neurotransmitters, inflammatory indicators, and endocrine functions leading to cognitive dysfunction and mood disorders. However, probiotics treatment ameliorates cognitive dysfunction and mood disorders. There is lack of clinical study to delineate the role of brain-gut axis in AUD induced cognitive and mood disorders in human. Therefore, a multi-center clinical studies with large-sample size is required to further elucidate the effects of alcohol on the brain-gut axis and therapeutic effects of probiotics. However, current preclinical studies show great potential. Therefore, we believe that therapeutic strategies targeting the gut microbiome will provide safe, novel and effective treatments for neuropsychiatric diseases.

## Author Contributions

XL, L-MC, and GK participated in data collection and writing the manuscript. S-JZ, Q-HZ, H-YZ, GG, and L-LW performed the literature search and screened titles and abstracts for their relevance to the objective of this work. H-ZF and J-WS designed, supervised the study, and revised the manuscript. All authors read and approved the final manuscript.

## Conflict of Interest

The authors declare that the research was conducted in the absence of any commercial or financial relationships that could be construed as a potential conflict of interest.

## Publisher’s Note

All claims expressed in this article are solely those of the authors and do not necessarily represent those of their affiliated organizations, or those of the publisher, the editors and the reviewers. Any product that may be evaluated in this article, or claim that may be made by its manufacturer, is not guaranteed or endorsed by the publisher.

## References

[B1] aan het Rot M. Mathew S. J. Charney D. S. (2009). Neurobiological mechanisms in major depressive disorder. *Cmaj* 180 305–313. 10.1503/cmaj.080697 19188629 PMC2630359

[B2] Adikari A. Appukutty M. Kuan G. (2020). Effects of daily probiotics supplementation on anxiety induced physiological parameters among competitive football players. *Nutrients* 12:71920. 10.3390/nu12071920 32610465 PMC7399934

[B3] Akira S. Hemmi H. (2003). Recognition of pathogen-associated molecular patterns by TLR family. *Immunol. Lett.* 85 85–95. 10.1016/s0165-2478(02)00228-6 12527213

[B4] Akkasheh G. Kashani-Poor Z. Tajabadi-Ebrahimi M. Jafari P. Akbari H. Taghizadeh M. (2016). Clinical and metabolic response to probiotic administration in patients with major depressive disorder: a randomized, double-blind, placebo-controlled trial. *Nutrition* 32 315–320. 10.1016/j.nut.2015.09.003 26706022

[B5] Alba-Ferrara L. Muller-Oehring E. M. Sullivan E. V. Pfefferbaum A. Schulte T. (2016). Brain responses to emotional salience and reward in alcohol use disorder. *Brain Imag. Behav.* 10 136–146. 10.1007/s11682-015-9374-8 25875013 PMC4607555

[B6] Aloi J. Blair K. S. Crum K. I. Meffert H. White S. F. Tyler P. M. (2018). Adolescents show differential dysfunctions related to Alcohol and Cannabis Use Disorder severity in emotion and executive attention neuro-circuitries. *Neuroimage Clin.* 19 782–792. 10.1016/j.nicl.2018.06.005 29988822 PMC6031867

[B7] Ames N. J. Barb J. J. Schuebel K. Mudra S. Meeks B. K. Tuason R. T. S. (2020). Longitudinal gut microbiome changes in alcohol use disorder are influenced by abstinence and drinking quantity. *Gut. Microbes.* 11 1608–1631. 10.1080/19490976.2020.1758010 32615913 PMC7527072

[B8] Bailey M. T. Dowd S. E. Galley J. D. Hufnagle A. R. Allen R. G. Lyte M. (2011). Exposure to a social stressor alters the structure of the intestinal microbiota: implications for stressor-induced immunomodulation. *Brain Behav. Immun.* 25 397–407. 10.1016/j.bbi.2010.10.023 21040780 PMC3039072

[B9] Bajaj J. S. (2019). Alcohol, liver disease and the gut microbiota. *Nat. Rev. Gastroenterol. Hepatol.* 16 235–246. 10.1038/s41575-018-0099-1 30643227

[B10] Barrett E. Ross R. P. O’Toole P. W. Fitzgerald G. F. Stanton C. (2012). gamma-Aminobutyric acid production by culturable bacteria from the human intestine. *J. Appl. Microbiol.* 113 411–417. 10.1111/j.1365-2672.2012.05344.x 22612585

[B11] Beck A. Wüstenberg T. Genauck A. Wrase J. Schlagenhauf F. Smolka M. N. (2012). Effect of brain structure, brain function, and brain connectivity on relapse in alcohol-dependent patients. *Arch. Gen. Psychiatry* 69 842–852. 10.1001/archgenpsychiatry.2011.2026 22868938

[B12] Bellono N. W. Bayrer J. R. Leitch D. B. Castro J. Zhang C. O’Donnell T. A. (2017). Enterochromaffin cells are gut chemosensors that couple to sensory neural pathways. *Cell* 18:e116. 10.1016/j.cell.2017.05.034 28648659 PMC5839326

[B13] Bercik P. Denou E. Collins J. Jackson W. Lu J. Jury J. (2011). The intestinal microbiota affect central levels of brain-derived neurotropic factor and behavior in mice. *Gastroenterology* 141 599–609. 10.1053/j.gastro.2011.04.052 21683077

[B14] Bercik P. Verdu E. F. Foster J. A. Macri J. Potter M. Huang X. (2010). Chronic gastrointestinal inflammation induces anxiety-like behavior and alters central nervous system biochemistry in mice. *Gastroenterology* 139 2102–2112. 10.1053/j.gastro.2010.06.063 20600016

[B15] Bercury K. K. Macklin W. B. (2015). Dynamics and mechanisms of CNS myelination. *Dev. Cell.* 32 447–458. 10.1016/j.devcel.2015.01.016 25710531 PMC6715306

[B16] Berridge C. W. Waterhouse B. D. (2003). The locus coeruleus-noradrenergic system: modulation of behavioral state and state-dependent cognitive processes. *Brain Res. Rev.* 42 33–84. 10.1016/s0165-0173(03)00143-7 12668290

[B17] Blakemore S. J. (2008). The social brain in adolescence. *Nat. Rev. Neurosci.* 9 267–277. 10.1038/nrn2353 18354399

[B18] Bohorquez D. V. Shahid R. A. Erdmann A. Kreger A. M. Wang Y. Calakos N. (2015). Neuroepithelial circuit formed by innervation of sensory enteroendocrine cells. *J. Clin. Invest.* 125 782–786. 10.1172/JCI78361 25555217 PMC4319442

[B19] Bonaz B. Bazin T. Pellissier S. (2018). The vagus nerve at the interface of the microbiota-gut-brain axis. *Front. Neurosci.* 12:49. 10.3389/fnins.2018.00049 29467611 PMC5808284

[B20] Bowden S. C. McCarter R. J. (1993). Spatial memory in alcohol-dependent subjects: using a push-button maze to test the principle of equiavailability. *Brain Cogn.* 22 51–62. 10.1006/brcg.1993.1024 8499112

[B21] Bravo J. A. Forsythe P. Chew M. V. Escaravage E. Savignac H. M. Dinan T. G. (2011). Ingestion of Lactobacillus strain regulates emotional behavior and central GABA receptor expression in a mouse *via* the vagus nerve. *Proc. Natl. Acad. Sci. USA* 108 16050–16055. 10.1073/pnas.1102999108 21876150 PMC3179073

[B22] Bruce S. E. Yonkers K. A. Otto M. W. Eisen J. L. Weisberg R. B. Pagano M. (2005). Influence of psychiatric comorbidity on recovery and recurrence in generalized anxiety disorder, social phobia, and panic disorder: a 12-year prospective study. *Am. J. Psychiatry* 162 1179–1187. 10.1176/appi.ajp.162.6.1179 15930067 PMC3272761

[B23] Bus B. A. Molendijk M. L. Tendolkar I. Penninx B. W. Prickaerts J. Elzinga B. M. (2015). Chronic depression is associated with a pronounced decrease in serum brain-derived neurotrophic factor over time. *Mol. Psychiatry* 20 602–608. 10.1038/mp.2014.83 25155878

[B24] Cai Y. Q. Wang W. Paulucci-Holthauzen A. Pan Z. Z. (2018). Brain circuits mediating opposing effects on emotion and pain. *J. Neurosci.* 38 6340–6349. 10.1523/JNEUROSCI.2780-17.2018 29941444 PMC6041794

[B25] Caldwell L. C. Schweinsburg A. D. Nagel B. J. Barlett V. C. Brown S. A. Tapert S. F. (2005). Gender and adolescent alcohol use disorders on BOLD (blood oxygen level dependent) response to spatial working memory. *Alcohol* 40 194–200. 10.1093/alcalc/agh134 15668210 PMC2270703

[B26] Camilleri M. (2009). Serotonin in the gastrointestinal tract. *Curr. Opin. Endocrinol. Diab. Obes.* 16 53–59. 10.1097/med.0b013e32831e9c8e 19115522 PMC2694720

[B27] Cerqueira J. J. Mailliet F. Almeida O. F. Jay T. M. Sousa N. (2007). The prefrontal cortex as a key target of the maladaptive response to stress. *J. Neurosci.* 27 2781–2787. 10.1523/JNEUROSCI.4372-06.2007 17360899 PMC6672565

[B28] Chandler C. M. Follett M. E. Porter N. J. Liang K. Y. Vallender E. J. Miller G. M. (2017). Persistent negative effects of alcohol drinking on aspects of novelty-directed behavior in male rhesus macaques. *Alcohol* 63 19–26. 10.1016/j.alcohol.2017.03.002 28847378 PMC5584881

[B29] Chen Y. H. Bai J. Wu D. Yu S. F. Qiang X. L. Bai H. (2019). Association between fecal microbiota and generalized anxiety disorder: severity and early treatment response. *J. Affect Disord.* 259 56–66. 10.1016/j.jad.2019.08.014 31437702

[B30] Chong H. X. Yusoff N. A. A. Hor Y. Y. Lew L. C. Jaafar M. H. Choi S. B. (2019). Lactobacillus plantarum DR7 alleviates stress and anxiety in adults: a randomised, double-blind, placebo-controlled study. *Benef. Microbes* 10 355–373. 10.3920/BM2018.0135 30882244

[B31] Clarke G. Grenham S. Scully P. Fitzgerald P. Moloney R. D. Shanahan F. (2013). The microbiome-gut-brain axis during early life regulates the hippocampal serotonergic system in a sex-dependent manner. *Mol. Psychiatry* 18 666–673. 10.1038/mp.2012.77 22688187

[B32] Clarke G. Stilling R. M. Kennedy P. J. Stanton C. Cryan J. F. Dinan T. G. (2014). Minireview: Gut microbiota: the neglected endocrine organ. *Mol. Endocrinol.* 28 1221–1238. 10.1210/me.2014-1108 24892638 PMC5414803

[B33] Contreras A. Polin E. Miguens M. Perez-Garcia C. Perez V. Ruiz-Gayo M. (2019). Intermittent-excessive and chronic-moderate ethanol intake during adolescence impair spatial learning, memory and cognitive flexibility in the adulthood. *Neuroscience* 418 205–217. 10.1016/j.neuroscience.2019.08.051 31491502

[B34] Correia S. S. Goosens K. A. (2016). Input-specific contributions to valence processing in the amygdala. *Learn Mem.* 23 534–543. 10.1101/lm.037887.114 27634144 PMC5026206

[B35] Crean R. D. Vandewater S. A. Katner S. N. Huitron-Resendiz S. Taffe M. A. (2011). Chronic alcohol consumption impairs visuo-spatial associative memory in periadolescent rhesus monkeys. *Drug Alcohol Depend.* 114 31–40. 10.1016/j.drugalcdep.2010.09.002 20951512 PMC3024459

[B36] Crews F. T. Bechara R. Brown L. A. Guidot D. M. Mandrekar P. Oak S. (2006). Cytokines and alcohol. *Alcohol Clin. Exp. Res.* 30 720–730. 10.1111/j.1530-0277.2006.00084.x 16573591

[B37] Cryan J. F. Dinan T. G. (2012). Mind-altering microorganisms: the impact of the gut microbiota on brain and behaviour. *Nat. Rev. Neurosci.* 13 701–712. 10.1038/nrn3346 22968153

[B38] Cryan J. F. Kaupmann K. (2005). Don’t worry ‘B’ happy!: a role for GABA(B) receptors in anxiety and depression. *Trends Pharmacol. Sci.* 26 36–43. 10.1016/j.tips.2004.11.004 15629203

[B39] Cussotto S. Sandhu K. V. Dinan T. G. Cryan J. F. (2018). The neuroendocrinology of the microbiota-gut-brain axis: a behavioural perspective. *Front. Neuroendocrinol.* 51 80–101. 10.1016/j.yfrne.2018.04.002 29753796

[B40] Dalile B. Van Oudenhove L. Vervliet B. Verbeke K. (2019). The role of short-chain fatty acids in microbiota-gut-brain communication. *Nat. Rev. Gastroenterol. Hepatol.* 16 461–478. 10.1038/s41575-019-0157-3 31123355

[B41] Dantzer R. O’Connor J. C. Lawson M. A. Kelley K. W. (2011). Inflammation-associated depression: from serotonin to kynurenine. *Psychoneuroendocrinology* 36 426–436. 10.1016/j.psyneuen.2010.09.012 21041030 PMC3053088

[B42] Desbonnet L. Clarke G. Shanahan F. Dinan T. G. Cryan J. F. (2014). Microbiota is essential for social development in the mouse. *Mol. Psychiatry* 19 146–148. 10.1038/mp.2013.65 23689536 PMC3903109

[B43] Desbonnet L. Clarke G. Traplin A. O’Sullivan O. Crispie F. Moloney R. D. (2015). Gut microbiota depletion from early adolescence in mice: Implications for brain and behaviour. *Brain Behav. Immun.* 48 165–173. 10.1016/j.bbi.2015.04.004 25866195

[B44] Diaz Heijtz R. Wang S. Anuar F. Qian Y. Björkholm B. Samuelsson A. (2011). Normal gut microbiota modulates brain development and behavior. *Proc. Natl. Acad. Sci. USA* 108 3047–3052. 10.1073/pnas.1010529108 21282636 PMC3041077

[B45] Erny D. Hrabe de Angelis A. L. Jaitin D. Wieghofer P. Staszewski O. David E. (2015). Host microbiota constantly control maturation and function of microglia in the CNS. *Nat. Neurosci.* 18 965–977. 10.1038/nn.4030 26030851 PMC5528863

[B46] Evrensel A. Unsalver B. O. Ceylan M. E. (2020). Neuroinflammation, gut-brain axis and depression. *Psychiatry Investig.* 17 2–8. 10.30773/pi.2019.08.09 31587531 PMC6992852

[B47] Fan Y. Ya E. Z. Ji-Dong W. Yu-Fan L. Ying Z. Ya-Lun S. (2018). Comparison of microbial diversity and composition in jejunum and colon of the alcohol-dependent rats. *J. Microbiol. Biotechnol.* 28 1883–1895. 10.4014/jmb.1806.06050 30270610

[B48] Faria R. Santana M. M. Aveleira C. A. Simoes C. Maciel E. Melo T. (2014). Alterations in phospholipidomic profile in the brain of mouse model of depression induced by chronic unpredictable stress. *Neuroscience* 273 1–11. 10.1016/j.neuroscience.2014.04.042 24814727

[B49] Gareau M. G. Wine E. Rodrigues D. M. Cho J. H. Whary M. T. Philpott D. J. (2011). Bacterial infection causes stress-induced memory dysfunction in mice. *Gut* 60 307–317. 10.1136/gut.2009.202515 20966022

[B50] Gass J. T. McGonigal J. T. Chandler L. J. (2017). Deficits in the extinction of ethanol-seeking behavior following chronic intermittent ethanol exposure are attenuated with positive allosteric modulation of mGlu5. *Neuropharmacology* 113 198–205. 10.1016/j.neuropharm.2016.10.005 27725153 PMC5148675

[B51] Gill S. R. Pop M. Deboy R. T. Eckburg P. B. Turnbaugh P. J. Samuel B. S. (2006). Metagenomic analysis of the human distal gut microbiome. *Science* 312 1355–1359. 10.1126/science.1124234 16741115 PMC3027896

[B52] Gimenez-Gomez P. Perez-Hernandez M. O’Shea E. Caso J. R. Martin-Hernandez D. Cervera L. A. (2019). Changes in brain kynurenine levels *via* gut microbiota and gut-barrier disruption induced by chronic ethanol exposure in mice. *FASEB J.* 33 12900–12914. 10.1096/fj.201900491RR 31509716 PMC6902706

[B53] Goh K. K. Liu Y. W. Kuo P. H. Chung Y. E. Lu M. L. Chen C. H. (2019). Effect of probiotics on depressive symptoms: A meta-analysis of human studies. *Psychiatry Res.* 282:112568. 10.1016/j.psychres.2019.112568 31563280

[B54] Gribble F. M. Reimann F. (2016). Enteroendocrine cells: chemosensors in the intestinal epithelium. *Annu. Rev. Physiol.* 78 277–299. 10.1146/annurev-physiol-021115-105439 26442437

[B55] Grill H. J. Hayes M. R. (2012). Hindbrain neurons as an essential hub in the neuroanatomically distributed control of energy balance. *Cell Metab.* 16 296–309. 10.1016/j.cmet.2012.06.015 22902836 PMC4862653

[B56] Gronier B. Savignac H. M. Di Miceli M. Idriss S. M. Tzortzis G. Anthony D. (2018). Increased cortical neuronal responses to NMDA and improved attentional set-shifting performance in rats following prebiotic (B-GOS((R))) ingestion. *Eur. Neuropsychopharmacol.* 28 211–224. 10.1016/j.euroneuro.2017.11.001 29174530 PMC5857269

[B57] Guida F. Turco F. Iannotta M. De Gregorio D. Palumbo I. Sarnelli G. (2018). Antibiotic-induced microbiota perturbation causes gut endocannabinoidome changes, hippocampal neuroglial reorganization and depression in mice. *Brain Behav. Immun.* 67 230–245. 10.1016/j.bbi.2017.09.001 28890155

[B58] Hachem L. D. Wong S. M. Ibrahim G. M. (2018). The vagus afferent network: emerging role in translational connectomics. *Neurosurg. Focus* 45:E2. 10.3171/2018.6.Focus18216 30173606

[B59] Heiss C. N. Olofsson L. E. (2018). Gut microbiota-dependent modulation of energy metabolism. *J. Innate. Immun.* 10 163–171. 10.1159/000481519 29131106 PMC6757175

[B60] Hermann A. Keck T. Stark R. (2014). Dispositional cognitive reappraisal modulates the neural correlates of fear acquisition and extinction. *Neurobiol. Learn Mem.* 113 115–124. 10.1016/j.nlm.2014.03.008 24713451

[B61] Hillemacher T. Bachmann O. Kahl K. G. Frieling H. (2018). Alcohol, microbiome, and their effect on psychiatric disorders. *Prog. Neuropsychopharmacol. Biol. Psychiatry* 85 105–115. 10.1016/j.pnpbp.2018.04.015 29705711

[B62] Holzer P. Reichmann F. Farzi A. (2012). Neuropeptide Y, peptide YY and pancreatic polypeptide in the gut-brain axis. *Neuropeptides* 46 261–274. 10.1016/j.npep.2012.08.005 22979996 PMC3516703

[B63] Hou X. Rong C. Wang F. Liu X. Sun Y. Zhang H. T. (2020). GABAergic system in stress: implications of GABAergic neuron subpopulations and the gut-vagus-brain pathway. *Neural. Plast.* 2020:8858415. 10.1155/2020/8858415 32802040 PMC7416252

[B64] Hsiao E. Y. McBride S. W. Hsien S. Sharon G. Hyde E. R. McCue T. (2013). Microbiota modulate behavioral and physiological abnormalities associated with neurodevelopmental disorders. *Cell* 155 1451–1463. 10.1016/j.cell.2013.11.024 24315484 PMC3897394

[B65] Hueston C. M. Cryan J. F. Nolan Y. M. (2017). Stress and adolescent hippocampal neurogenesis: diet and exercise as cognitive modulators. *Transl. Psychiatry* 7:e1081. 10.1038/tp.2017.48 28375209 PMC5416690

[B66] Hwang Y. H. Park S. Paik J. W. Chae S. W. Kim D. H. Jeong D. G. (2019). Efficacy and safety of Lactobacillus plantarum C29-fermented soybean (DW2009) in individuals with mild cognitive impairment: a 12-week, multi-center, randomized, double-blind, placebo-controlled clinical trial[J]. *Nutrients* 11:305. 10.3390/nu11020305 30717153 PMC6412773

[B67] Jadhav K. S. Peterson V. L. Halfon O. Ahern G. Fouhy F. Stanton C. (2018). Gut microbiome correlates with altered striatal dopamine receptor expression in a model of compulsive alcohol seeking. *Neuropharmacology* 141 249–259. 10.1016/j.neuropharm.2018.08.026 30172845

[B68] Jansen J. M. Holst R. V. Wim V. Veltman D. J. Ca An M. Biology A. G. J. A. (2015). Brain function during cognitive flexibility and white matter integrity in alcohol-dependent patients, problematic drinkers and healthy controls. *Addict Biol.* 20 979–989. 25477246 10.1111/adb.12199

[B69] Jiang H. Ling Z. Zhang Y. Mao H. Ma Z. Yin Y. (2015). Altered fecal microbiota composition in patients with major depressive disorder. *Brain Behav. Immun.* 48 186–194. 10.1016/j.bbi.2015.03.016 25882912

[B70] Kaelberer M. M. Buchanan K. L. Klein M. E. Barth B. B. Montoya M. M. Shen X. (2018). A gut-brain neural circuit for nutrient sensory transduction. *Science* 361 5236. 10.1126/science.aat5236 30237325 PMC6417812

[B71] KobayashiY.KinoshitaT.MatsumotoA.YoshinoK.SaitoI.XiaoJ. Z. (2019a). Bifidobacterium Breve A1 supplementation improved cognitive decline in older adults with mild cognitive impairment: an open-label, single-arm study. *J. Prev. Alzheimers Dis.* 6 70–75. 10.14283/jpad.2018.32 30569089

[B72] Kobayashi Y. Kuhara T. Oki M. Xiao J. Z. (2019b). Effects of Bifidobacterium breve A1 on the cognitive function of older adults with memory complaints: a randomised, double-blind, placebo-controlled trial. *Benef. Microbes* 10 511–520. 10.3920/BM2018.0170 31090457

[B73] Kraynak T. E. Marsland A. L. Wager T. D. Gianaros P. J. (2018). Functional neuroanatomy of peripheral inflammatory physiology: a meta-analysis of human neuroimaging studies. *Neurosci. Biobehav. Rev.* 94 76–92. 10.1016/j.neubiorev.2018.07.013 30067939 PMC6363360

[B74] Kumar K. Sharma S. Kumar P. Deshmukh R. (2013). Therapeutic potential of GABA(B) receptor ligands in drug addiction, anxiety, depression and other CNS disorders. *Pharmacol. Biochem. Behav.* 110 174–184. 10.1016/j.pbb.2013.07.003 23872369

[B75] Kushner M. G. Abrams K. Borchardt C. (2000). The relationship between anxiety disorders and alcohol use disorders: a review of major perspectives and findings. *Clin. Psychol. Rev.* 20 149–171. 10.1016/s0272-7358(99)00027-6 10721495

[B76] Leclercq S. Matamoros S. Cani P. D. Neyrinck A. M. Jamar F. Starkel P. (2014). Intestinal permeability, gut-bacterial dysbiosis, and behavioral markers of alcohol-dependence severity. *Proc. Natl. Acad. Sci. USA* 111 E4485–E4493. 10.1073/pnas.1415174111 25288760 PMC4210345

[B77] Leclercq S. Schwarz M. Delzenne N. M. Starkel P. de Timary P. (2021). Alterations of kynurenine pathway in alcohol use disorder and abstinence: a link with gut microbiota, peripheral inflammation and psychological symptoms. *Transl. Psychiatry* 11:503. 10.1038/s41398-021-01610-5 34599147 PMC8486842

[B78] Liappas I. Theotoka I. Kapaki E. Ilias I. Paraskevas G. P. Soldatos C. R. (2007). Neuropsychological assessment of cognitive function in chronic alcohol-dependent patients and patients with Alzheimer’s disease. *Vivo* 21 1115–1118. 18210766

[B79] Liu S. (2016). The development of our organ of other kinds-the gut microbiota. *Front. Microbiol.* 7:2107. 10.3389/fmicb.2016.02107 28066404 PMC5179505

[B80] Luczynski P. McVey Neufeld K. A. Oriach C. S. Clarke G. Dinan T. G. Cryan J. F. (2016a). Growing up in a bubble: using germ-free animals to assess the influence of the gut microbiota on brain and behavior. *Int. J. Neuropsychopharmacol.* 19:20. 10.1093/ijnp/pyw020 26912607 PMC5006193

[B81] Luczynski P. Whelan S. O. O’Sullivan C. Clarke G. Shanahan F. Dinan T. G. (2016b). Adult microbiota-deficient mice have distinct dendritic morphological changes: differential effects in the amygdala and hippocampus. *Eur. J. Neurosci.* 44 2654–2666. 10.1111/ejn.13291 27256072 PMC5113767

[B82] Luo J. Wang T. Liang S. Hu X. Li W. Jin F. (2014). Ingestion of Lactobacillus strain reduces anxiety and improves cognitive function in the hyperammonemia rat. *Sci. China Life Sci.* 57 327–335. 10.1007/s11427-014-4615-4 24554471

[B83] Macfarlane G. T. Macfarlane S. (2012). Bacteria, colonic fermentation, and gastrointestinal health. *J. AOAC Int.* 95 50–60. 10.5740/jaoacint.sge_macfarlane 22468341

[B84] Ma C. Zhang C. Chen D. Jiang S. Shen S. Huo D. (2021). Probiotic consumption influences universal adaptive mutations in indigenous human and mouse gut microbiota. *Commun. Biol.* 4:1198. 10.1038/s42003-021-02724-8 34663913 PMC8523657

[B85] Manning V. Teo H. C. Guo S. Wong K. E. Li T. K. (2016). Neurocognitive functioning and treatment outcome following detoxification among asian alcohol-dependent inpatients. *Subst. Use Misuse.* 51 193–205. 10.3109/10826084.2015.1092985 26771240

[B86] Marin M. F. Zsido R. G. Song H. Lasko N. B. Killgore W. D. S. Rauch S. L. (2017). Skin conductance responses and neural activations during fear conditioning and extinction recall across anxiety disorders. *JAMA Psychiatry* 74 622–631. 10.1001/jamapsychiatry.2017.0329 28403387 PMC5539837

[B87] Mawdsley J. E. Rampton D. S. (2005). Psychological stress in IBD: new insights into pathogenic and therapeutic implications. *Gut* 54 1481–1491. 10.1136/gut.2005.064261 16162953 PMC1774724

[B88] Miyaoka T. Kanayama M. Wake R. Hashioka S. Hayashida M. Nagahama M. (2018). Clostridium butyricum MIYAIRI 588 as adjunctive therapy for treatment-resistant major depressive disorder: a prospective open-label trial. *Clin. Neuropharmacol.* 41 151–155. 10.1097/WNF.0000000000000299 30234616

[B89] Mohle L. Mattei D. Heimesaat M. M. Bereswill S. Fischer A. Alutis M. (2016). Ly6C(hi) Monocytes provide a link between antibiotic-induced changes in gut microbiota and adult hippocampal neurogenesis. *Cell Rep.* 15 1945–1956. 10.1016/j.celrep.2016.04.074 27210745

[B90] Moloney R. D. Desbonnet L. Clarke G. Dinan T. G. Cryan J. F. (2014). The microbiome: stress, health and disease. *Mamm. Genome* 25 49–74. 10.1007/s00335-013-9488-5 24281320

[B91] Nagahara A. H. Otto T. Gallagher M. (1995). Entorhinal-perirhinal lesions impair performance of rats on two versions of place learning in the Morris water maze. *Behav. Neurosci.* 109, 3–9. 10.1037//0735-7044.109.1.3 7734077

[B92] Nikolova V. L. Hall M. R. B. Hall L. J. Cleare A. J. Stone J. M. Young A. H. (2021). Perturbations in gut microbiota composition in psychiatric disorders: a review and meta-analysis. *JAMA Psychiatry.* 78 1343–1354. 10.1001/jamapsychiatry.2021.2573 34524405 PMC8444066

[B93] Ng Q. X. Peters C. Ho C. Y. X. Lim D. Y. Yeo W. S. (2018). A meta-analysis of the use of probiotics to alleviate depressive symptoms. *J. Affect. Disord.* 228 13–19. 10.1016/j.jad.2017.11.063 29197739

[B94] O’Brien S. M. Scott L. V. Dinan T. G. (2004). Cytokines: abnormalities in major depression and implications for pharmacological treatment. *Hum. Psychopharmacol.* 19 397–403. 10.1002/hup.609 15303243

[B95] O’Connor J. C. Lawson M. A. Andre C. Briley E. M. Szegedi S. S. Lestage J. (2009). Induction of IDO by bacille Calmette-Guerin is responsible for development of murine depressive-like behavior. *J. Immunol.* 182 3202–3212. 10.4049/jimmunol.0802722 19234218 PMC2664258

[B96] O’Mahony S. M. Clarke G. Dinan T. G. Cryan J. F. (2017). Early-life adversity and brain development: Is the microbiome a missing piece of the puzzle? *Neuroscience* 342 37–54. 10.1016/j.neuroscience.2015.09.068 26432952

[B97] Oliveira T. G. Chan R. B. Bravo F. V. Miranda A. Silva R. R. Zhou B. (2016). The impact of chronic stress on the rat brain lipidome. *Mol. Psychiatry* 21 80–88. 10.1038/mp.2015.14 25754084 PMC4565780

[B98] Olivier J. D. Vinkers C. H. Olivier B. (2013). The role of the serotonergic and GABA system in translational approaches in drug discovery for anxiety disorders. *Front. Pharmacol.* 4:74. 10.3389/fphar.2013.00074 23781201 PMC3677985

[B99] Orhan F. Bhat M. Sandberg K. Stahl S. Piehl F. Karolinska Schizophrenia Project (2016). Tryptophan metabolism along the kynurenine pathway downstream of toll-like receptor stimulation in peripheral monocytes. *Scand. J. Immunol.* 84 262–271. 10.1111/sji.12479 27607184

[B100] Oscar-Berman M. Valmas M. M. Sawyer K. S. Ruiz S. M. Luhar R. B. Gravitz Z. R. (2014). Profiles of impaired, spared, and recovered neuropsychologic processes in alcoholism. *Handb. Clin. Neurol.* 125 183–210. 10.1016/B978-0-444-62619-6.00012-4 25307576 PMC4515358

[B101] Petra A. I. Panagiotidou S. Hatziagelaki E. Stewart J. M. Conti P. Theoharides T. C. (2015). Gut-Microbiota-brain axis and its effect on neuropsychiatric disorders with suspected immune dysregulation. *Clin. Ther.* 37 984–995. 10.1016/j.clinthera.2015.04.002 26046241 PMC4458706

[B102] Peirce J. M. Alviña K. (2019). The role of inflammation and the gut microbiome in depression and anxiety. *J. Neurosci. Res.* 97 1223–1241. 10.1002/jnr.24476 31144383

[B103] Pinto-Sanchez M. I. Hall G. B. Ghajar K. Nardelli A. Bolino C. Lau J. T. (2017). Probiotic Bifidobacterium longum NCC3001 reduces depression scores and alters brain activity: a pilot study in patients with irritable bowel syndrome. *Gastroenterology* 153 448–459. 10.1053/j.gastro.2017.05.003 28483500

[B104] Roshchina V. V. (2016). New trends and perspectives in the evolution of neurotransmitters in microbial. Plant, and Animal Cells. *Adv. Exp. Med. Biol.* 874 25–77. 10.1007/978-3-319-20215-0_2 26589213

[B105] Rudzki L. Ostrowska L. Pawlak D. Malus A. Pawlak K. Waszkiewicz N. (2019). Probiotic Lactobacillus Plantarum 299v decreases kynurenine concentration and improves cognitive functions in patients with major depression: a double-blind, randomized, placebo controlled study. *Psychoneuroendocrinology* 100 213–222. 10.1016/j.psyneuen.2018.10.010 30388595

[B106] Santin L. J. Rubio S. Begega A. Arias J. L. (2000). Effects of chronic alcohol consumption on spatial reference and working memory tasks. *Alcohol* 20 149–159. 10.1016/s0741-8329(99)00070-1 10719794

[B107] Savignac H. M. Kiely B. Dinan T. G. Cryan J. F. (2014). Bifidobacteria exert strain-specific effects on stress-related behavior and physiology in BALB/c mice. *Neurogastroenterol. Motil.* 26 1615–1627. 10.1111/nmo.12427 25251188

[B108] Schulte T. Oberlin B. G. Kareken D. A. Marinkovic K. Muller-Oehring E. M. Meyerhoff D. J. (2012). How acute and chronic alcohol consumption affects brain networks: insights from multimodal neuroimaging. *Alcohol Clin. Exp. Res.* 36 2017–2027. 10.1111/j.1530-0277.2012.01831.x 22577873 PMC4500115

[B109] Schroeder F. A. Lin C. L. Crusio W. E. Akbarian S. (2007). Antidepressant-like effects of the histone deacetylase inhibitor, sodium butyrate, in the mouse. *Biol. Psychiatry* 62 55–64. 10.1016/j.biopsych.2006.06.036 16945350

[B110] Schwarzmeier H. Kleint N. I. Wittchen H. U. Strohle A. Hamm A. O. Lueken U. (2019). Characterizing the nature of emotional-associative learning deficits in panic disorder: An fMRI study on fear conditioning, extinction training and recall. *Eur. Neuropsychopharmacol.* 29 306–318. 10.1016/j.euroneuro.2018.11.1108 30497840

[B111] Scott L. V. Clarke G. Dinan T. G. (2013). The brain-gut axis: a target for treating stress-related disorders. *Mod. Trends Pharmacopsychiatry* 28 90–99. 10.1159/000343971 25224893

[B112] Shallice T. Cipolotti L. (2018). The prefrontal cortex and neurological impairments of active thought. *Annu. Rev. Psychol.* 69 157–180. 10.1146/annurev-psych-010416-044123 28813204

[B113] Sharma A. N. Chopde C. T. Hirani K. Kokare D. M. Ugale R. R. (2007). Chronic progesterone treatment augments while dehydroepiandrosterone sulphate prevents tolerance to ethanol anxiolysis and withdrawal anxiety in rats. *Eur. J. Pharmacol.* 567 211–222. 10.1016/j.ejphar.2007.04.025 17511983

[B114] Sherwin E. Bordenstein S. R. Quinn J. L. Dinan T. G. Cryan J. F. (2019). Microbiota and the social brain. *Science* 366:2016. 10.1126/science.aar2016 31672864

[B115] Sgritta M. Dooling S. W. Buffington S. A. Momin E. N. Francis M. B. Britton R. A. (2019). Mechanisms underlying microbial-mediated changes in social behavior in mouse models of autism spectrum disorder. *Neuron* 101 246–259. 10.1016/j.neuron.2018.11.018 30522820 PMC6645363

[B116] Sripada C. S. Angstadt M. McNamara P. King A. C. Phan K. L. (2011). Effects of alcohol on brain responses to social signals of threat in humans. *Neuroimage* 55 371–380. 10.1016/j.neuroimage.2010.11.062 21122818 PMC3031714

[B117] Stasi C. Sadalla S. Milani S. (2019). The Relationship Between the Serotonin Metabolism. Gut-Microbiota and the Gut-Brain Axis. *Curr. Drug Metab.* 20 646–655. 10.2174/1389200220666190725115503 31345143

[B118] Stavro K. Pelletier J. Potvin S. J. A. B. (2013). Widespread and sustained cognitive deficits in alcoholism: a meta-analysis. *Addict. Biol.* 18 203–213. 22264351 10.1111/j.1369-1600.2011.00418.x

[B119] Stilling R. M. Dinan T. G. Cryan J. F. (2014). Microbial genes, brain & behaviour - epigenetic regulation of the gut-brain axis. *Genes Brain Behav.* 13 69–86. 10.1111/gbb.12109 24286462

[B120] Strandwitz P. Kim K. H. Terekhova D. Liu J. K. Sharma A. Levering J. (2019). GABA-modulating bacteria of the human gut microbiota. *Nat. Microbiol.* 4 396–403. 10.1038/s41564-018-0307-3 30531975 PMC6384127

[B121] Strandwitz P. (2018). Neurotransmitter modulation by the gut microbiota. *Brain Res.* 1693 128–133. 10.1016/j.brainres.2018.03.015 29903615 PMC6005194

[B122] Suarez A. N. Hsu T. M. Liu C. M. Noble E. E. Cortella A. M. Nakamoto E. M. (2018). Gut vagal sensory signaling regulates hippocampus function through multi-order pathways. *Nat. Commun.* 9:2181. 10.1038/s41467-018-04639-1 29872139 PMC5988686

[B123] Wang Y. Kasper L. H. (2014). The role of microbiome in central nervous system disorders. *Brain Behav. Immun.* 38 1–12. 10.1016/j.bbi.2013.12.015 24370461 PMC4062078

[B124] Wei J. Qin L. Fu Y. Dai Y. Wen Y. Xu S. (2019). Long-term consumption of alcohol exacerbates neural lesions by destroying the functional integrity of the blood-brain barrier. *Drug Chem. Toxicol.* 2019 1–8. 10.1080/01480545.2019.1681444 31746246

[B125] WHO (2014). *Global Status Report on Alcohol.* Available online at: https://www.who.int/news-room/fact-sheets/detail/e-coli [Accessed March 09, 2021]

[B126] Wichers M. C. Maes M. (2004). The role of indoleamine 2,3-dioxygenase (IDO) in the pathophysiology of interferon-alpha-induced depression. *J. Psychiatry Neurosci.* 29 11–17. 14719046 PMC305266

[B127] Wikoff W. R. Anfora A. T. Liu J. Schultz P. G. Lesley S. A. Peters E. C. (2009). Metabolomics analysis reveals large effects of gut microflora on mammalian blood metabolites. *Proc. Natl. Acad. Sci. USA* 106 3698–3703. 10.1073/pnas.0812874106 19234110 PMC2656143

[B128] Williams E. K. Chang R. B. Strochlic D. E. Umans B. D. Lowell B. B. Liberles S. D. (2016). Sensory neurons that detect stretch and nutrients in the digestive system. *Cell* 166 209–221. 10.1016/j.cell.2016.05.011 27238020 PMC4930427

[B129] Witkiewitz K. Litten R. Z. Leggio L. (2019). Advances in the science and treatment of alcohol use disorder. *Sci. Adv.* 5:eaax4043. 10.1126/sciadv.aax4043 31579824 PMC6760932

[B130] Xiao H. W. Ge C. Feng G. X. Li Y. Luo D. Dong J. L. (2018). Gut microbiota modulates alcohol withdrawal-induced anxiety in mice. *Toxicol. Lett.* 287 23–30. 10.1016/j.toxlet.2018.01.021 29391279 PMC5912338

[B131] Yano J. M. Yu K. Donaldson G. P. Shastri G. G. Ann P. Ma L. (2015). Indigenous bacteria from the gut microbiota regulate host serotonin biosynthesis. *Cell* 161 264–276. 10.1016/j.cell.2015.02.047 25860609 PMC4393509

[B132] Yeager M. P. Pioli P. A. Guyre P. M. (2011). Cortisol exerts bi-phasic regulation of inflammation in humans. *Dose. Response* 9 332–347. 10.2203/dose-response.10-013.Yeager 22013396 PMC3186928

[B133] Yu C. D. Xu Q. J. Chang R. B. (2020). Vagal sensory neurons and gut-brain signaling. *Curr. Opin. Neurobiol.* 62 133–140. 10.1016/j.conb.2020.03.006 32380360 PMC7560965

[B134] Yunes R. A. Poluektova E. U. Vasileva E. V. Odorskaya M. V. Marsova M. V. Kovalev G. I. (2020). A multi-strain potential probiotic formulation of GABA-producing lactobacillus plantarum 90sk and Bifidobacterium adolescentis 150 with Antidepressant Effects. *Probiot. Antimicrob. Prot.* 12 973–979. 10.1007/s12602-019-09601-1 31677091

[B135] Zhan X. Stamova B. Jin L. W. DeCarli C. Phinney B. Sharp F. R. (2016). Gram-negative bacterial molecules associate with Alzheimer disease pathology. *Neurology* 87 2324–2332. 10.1212/WNL.0000000000003391 27784770 PMC5135029

[B136] Zhao W. Hu Y. Li C. Li N. Zhu S. Tan X. (2020). Transplantation of fecal microbiota from patients with alcoholism induces anxiety/depression behaviors and decreases brain mGluR1/PKC epsilon levels in mouse. *Biofactors* 46 38–54. 10.1002/biof.1567 31518024

